# Weighted Beta Regression for Modeling Bounded Data

**DOI:** 10.3390/e28101119

**Published:** 2026-10-13

**Authors:** Marcelo Bourguignon, Diego I. Gallardo, Roberto Vila

**Affiliations:** 1 Department of Statistics, Universidade Federal do Rio Grande do Norte, Natal 59078-970, RN, Brazil; 2 Department of Statistics, Facultad de Ciencias, Universidad del Bío-Bío, Concepción 4081112, Chile; dgallardo@ubiobio.cl; 3 Department of Statistics, Universidade de Brasília, Brasília 70910-900, DF, Brazil; rovig161@gmail.com

**Keywords:** beta distribution, finite mixture model, proportions, regression, weighted version

## Abstract

A two-parameter weighted beta distribution is introduced for modeling bounded data, which has many similarities to the beta distribution. We propose a class of regression models where the response is weighted beta distributed and the two shape parameters that index the weighted beta distribution are related to covariates and regression parameters. The proposed regression model is a natural strong competitor of the beta regression model. We study the mathematical and statistical properties of the distribution, and we provide a useful interpretation of the parameters. The maximum likelihood method is used for estimating the model parameters. We conduct simulation studies to investigate the performance of the maximum likelihood estimators and the asymptotic confidence intervals of the parameters. We present an application of the proposed regression model to real bounded data.

## 1. Introduction

The two-parameter beta distribution is the most widely used model in the literature to describe continuous bounded data, especially because of its flexibility [1]. The random variable *B* follows a beta distribution with shape parameters α,β>0, denoted by B∼Beta(α,β), if its probability density function (PDF) is expressed as

$$f_{B}\left(x;\alpha ,\beta \right)=\frac{1}{B(\alpha ,\beta )}\;x^{\alpha -1}\;{\left(1-x\right)}^{\beta -1},\;0<x<1,$$

where $B\left(\alpha ,\beta \right)=\int_{0}^{1}\;t^{\alpha }\;{\left(1-t\right)}^{\beta -1}dt$ is the complete beta function.

Ref. [2] showed that if $G_{1}$ and $G_{2}$ are independent random variables following gamma distributions with shape parameters α>0 and β>0, and same scale parameter θ, then

(1) $$B\overset{d}{=}\frac{G_{1}}{G_{1}+G_{2}}∼\mathrm{Beta}\left(\alpha ,\beta \right),$$

namely, *B* is beta distributed with shape parameters α,β>0 and $\overset{d}{=}$ stands for equality in distribution. It should be noted that stochastic representations are important since they may justify some models arising naturally in real situations, as seen above. For a detailed discussion of the beta distribution, interested readers may refer to [1,3,4].

Recently, researchers have introduced new families of distributions to model bounded quantities using the beta distribution as an integral feature of the model. For example, ref. [5] introduced the complementary beta distribution. Ref. [6] studied the beta rectangular distribution. Ref. [7] considered the tilted beta distribution. To accommodate explanatory variables in modeling the above distributions, see [8], [9], and [7], respectively. However, to our knowledge, these alternatives do not have a stochastic representation analogous to (1).

Ref. [10] introduced the concept of weighted distribution. The concept of weighted distribution has been used to select the appropriate model for observed data, especially when samples are recorded without a sampling frame that enables random sampling. In fact, ref. [11] wrote: “Weighted distributions occur in a natural way in specifying probabilities of events as observed and recorded by making adjustments to probabilities of actual occurrence of events taking into account methods of ascertainment. Failure to make such adjustments can lead to wrong conclusions.” Many authors have discussed weighted distributions. However, to the best of our knowledge, a specific weighted model to describe data observed on the interval (0, 1) satisfying the stochastic representation such as (1) has never been considered in the literature.

In this context, the goal of this paper is to propose a new regression model based on the new weighted beta (WB) distribution and investigate it as a useful alternative to the beta regression model, with the hope that the new model may have a “better fit” in certain practical situations. The proposed model therefore allows one to study the relationship between the variable of interest and other variables that affect its behavior and can be used whenever the response is measured as a rate or as a proportion. Thus, one of the main motivations of this paper is to provide an attractive alternative regression model for bounded response variables. The main contributions and advantages of the proposed model are as follows:

- The proposed distribution has only two parameters, in contrast with some generalizations of the beta distribution which have three or four parameters.
- The proposed distribution of this process satisfies the stochastic representation given in (1) (see Theorem 2) by replacing the gamma assumption with the weighted Lindley (WL) distribution [12].
- The WB distribution can be viewed as a mixture of two beta distributions with “known” weights, in contrast to a mixture of beta distributions. The weights depend only on the parameters of the proposed distribution (see Proposition 3).
- The WB distribution is a discrete mixed Beta distribution [13] (see Corollary 2).
- Based on the WB distribution, we propose two new weighted distributions: weighted beta prime (WBP) and weighted Libby-Novick (WLN) distributions (see Proposition 4).
- We define a class of models based on the WB distribution where the two shape parameters that index the distribution have regression structures defined by sets of explanatory variables. We discuss maximum likelihood estimation of the regression parameters, obtaining the log-likelihood function, the score function, and the Hessian matrix. We also propose a simple interpretation of the predictor response relationship in terms of percentage increases/decreases in the logarithm of the odds ratio for the mixing probability (see Remark 3).
- The WB distribution can provide better fits than the beta distribution in some situations (see Section 7).

The rest of this paper proceeds as follows. In Section 2, we define the WB distribution and derive some of its properties. In Section 3, we analyze the stochastic and statistical properties of the WB distribution. Section 4 discusses the WB regression model and maximum likelihood estimation. Section 5 considers residuals and diagnostic measures. Section 6 presents numerical results for the estimators and the empirical distribution of the residuals. Section 7 presents an empirical illustration and its diagnostic analysis to demonstrate the usefulness of the proposed model. Section 8 presents concluding remarks.

## 2. The Weighted Beta Distribution

In this section, we formulate the new distribution and obtain some of its more relevant characteristics and some mathematical properties. Let *Z* be a random variable following a beta distribution with shape parameters α,β>0, and suppose that when Z=z occurs, the probability of recording it is w(z,α,β), depending on the observed value *z* and possibly also on unknown parameters α and β. Then, the PDF of the resulting random variable $Z^{w}$ is

(2) $$f_{Z}\left(z;\alpha ,\beta \right)=\frac{w\left(z,\alpha ,\beta \right)z^{\alpha -1}{\left(1-z\right)}^{\beta -1}}{B\left(\alpha ,\beta \right)E_{B}\left[w\left(Z,\alpha ,\beta \right)\right]},\;0<z<1,$$

where $E_{B}\left[w\left(Z,\alpha ,\beta \right)\right]$ denotes the expectation with respect to the beta distribution. In deriving (2), we may choose w(z,α,β) such that 0≤w(z,α,β)≤1. However, (2) can be defined for any nonnegative weight function for any arbitrary nonnegative weight function w(z,α,β) for which $E_{B}\left[w\left(Z,\alpha ,\beta \right)\right]$ exists. The distribution (2) obtained by using any nonnegative weight function w(z,α,β) is called a weighted version of *Z* [10]. In particular, when the weight function is 0≤w(z)=z≤1, which is the so-called size-biased version of the beta distribution. In this paper, we consider a particular nonnegative weight function w(z,α,β)=z(1−z)+1/(α+β). The choice of these weights is due to the fact that their use leads to excellent properties for the distribution (see Remark 1 and Proposition 1 below). Thus,

$$E_{B}\left[w\left(Z,\alpha ,\beta \right)\right]=E_{B}\left[Z\left(1-Z\right)+1/\left(\alpha +\beta \right)\right]=\frac{\left(\alpha +1)(\beta +1\right)}{\left(\alpha +\beta )(\alpha +\beta +1\right)}.$$

**Definition 1.** 
*The random variable Z follows a WB distribution with shape parameters α,β>0 if its PDF is expressed as*

(3) $$f_{Z}\left(z;\alpha ,\beta \right)=\frac{\left(\alpha +\beta +1\right)}{\left(1+\alpha )(1+\beta \right)}\;\frac{z^{\alpha -1}{\left(1-z\right)}^{\beta -1}}{B(\alpha ,\beta )}\left[(\alpha +\beta )\;z\left(1-z\right)+1\right],\;0<z<1.$$

A random variable *Z* with PDF (3) is denoted by Z∼WB(α,β). If Z∼WB(α,β), then its complement (1−Z) is WB(β,α) distributed. This property is similar to the one enjoyed by the beta distribution. The behaviors of the PDF of the weighted beta distribution $f_{Z}\left(z;\alpha ,\beta \right)$ at z=0 and z=1, respectively, are given by

$$\lim_{z\to 0^{+}}f_{Z}\left(z;\alpha ,\beta \right)=\left\{\begin{array}{cc} \infty , & \alpha <1,\\ \frac{\left(2+\beta \right)}{2(1+\beta )B(1,\beta )}, & \alpha =1,\\ 0, & \alpha >1, \end{array}\right.\;\lim_{z\to 1^{-}}f_{Z}\left(z;\alpha ,\beta \right)=\left\{\begin{array}{cc} \infty , & \beta <1,\\ \frac{\left(2+\alpha \right)}{2(1+\alpha )B(\alpha ,1)}, & \beta =1,\\ 0, & \beta >1. \end{array}\right.$$

**Figure 1:**
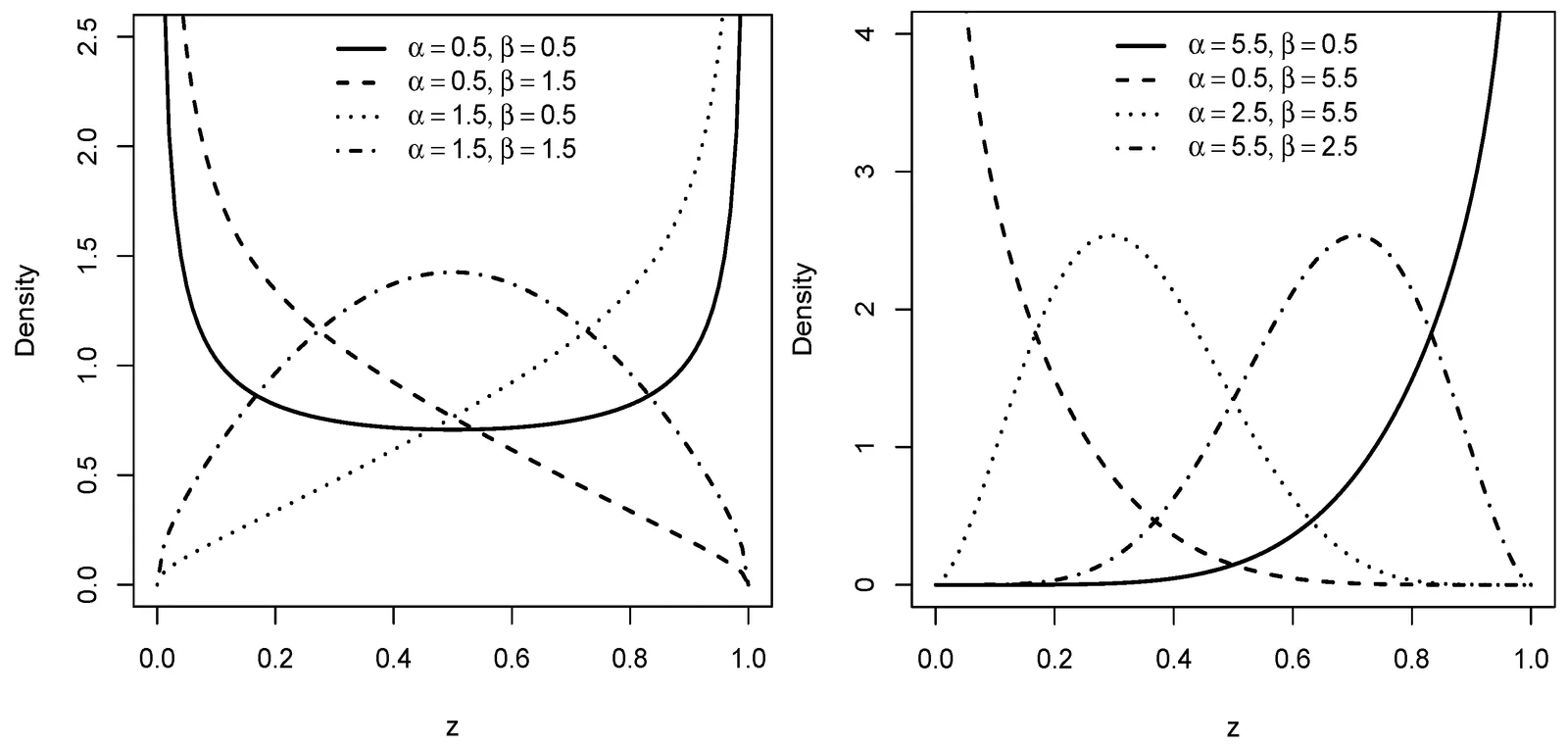
WB densities for different combinations of (α,β).

From Figure 1, we distinguish different shapes of the WB PDF for different parameter combinations. In the case that α=β=1, $f_{Z}\left(z;1,1\right)=3\left[2z\left(1-z\right)+1\right]/4,\;0<z<1,$ is unimodal with mode z=1/2. When α=1 and β≠1, $f_{Z}\left(z;1,\beta \right)=C\left(\beta \right){\left(1-z\right)}^{\beta -1}\left[(1+\beta )\;z\left(1-z\right)+1\right],\;0<z<1,$ with C(β)=(2+β)/[2(1+β)B(1,β)] and

$$\begin{array}{c} \frac{df_{Z}\left(z;1,\beta \right)}{dz}=C\left(\beta \right){\left(1-z\right)}^{\beta -2}p_{2}\left(z;\beta \right), \end{array}$$

where $p_{2}\left(z;\beta \right)$ is the quadratic polynomial defined as $p_{2}\left(z;\beta \right)={\left(1+\beta \right)}^{2}z^{2}-\left(1+\beta \right)\left(2+\beta \right)z+2.$ In other words, the number of critical points (if any) of the WB PDF $f_{Z}\left(z;1,\beta \right)$ is determined by the number of zeros of the polynomial $p_{2}\left(z;\beta \right)$. The following result follows from a simple analysis of the quadratic polynomial $p_{2}\left(z;\beta \right)$, so its proof will be omitted.

**Proposition 1.** 
*The following hold:*

*1.*  *If α=1 and $\beta <2(\sqrt{2}-1)$, then the WB PDF $z⟼f_{Z}\left(z;1,\beta \right)$ is increasing.*
*2.*  *If $\alpha =1>2(\sqrt{2}-1)=\beta$, then the WB PDF $z⟼f_{Z}\left(z;1,\beta \right)$ has a U-shape, with minimum point z=(2+β)/[2(1+β)].*
*3.*  *If $\alpha =1>\beta >2(\sqrt{2}-1)$, then the WB PDF $z⟼f_{Z}\left(z;1,\beta \right)$ is increasing-decreasing-increasing, with maximum point $z=\left(2+\beta -\sqrt{\beta^{2}+4\beta -4}\right)/\left[2\left(1+\beta \right)\right]$ and minimum point $z=\left(2+\beta +\sqrt{\beta^{2}+4\beta -4}\right)/\left[2\left(1+\beta \right)\right]$.*
*4.*  *If α=1<β, then the WB PDF $z⟼f_{Z}\left(z;1,\beta \right)$ is unimodal, with mode $z=\left(2+\beta -\sqrt{\beta^{2}+4\beta -4}\right)/\left[2\left(1+\beta \right)\right]$.*

Now, in the case that α≠1 and β=1, $f_{Z}\left(z;\alpha ,1\right)=C\left(\alpha \right)z^{\alpha -1}\left[\left(\alpha +1\right)\;z\left(1-z\right)+1\right],\;0<z<1,$ where C(α)=(α+2)/[2(1+α)B(α,1)] and

$$\begin{array}{c} \frac{df_{Z}\left(z;\alpha ,1\right)}{dz}=C\left(\alpha \right)z^{\alpha -2}p_{2}\left(z;\alpha \right), \end{array}$$

with $p_{2}\left(z;\alpha \right)$ being the quadratic polynomial defined as $p_{2}\left(z;\alpha \right)=-{\left(\alpha +1\right)}^{2}z^{2}+\alpha \left(\alpha +1\right)z+\alpha -1.$ The next result follows from a standard analysis of the polynomial $p_{2}\left(z;\alpha \right)$, so we decided to omit its proof.

**Proposition 2.** 
*The following hold:*

*1.*  *If $\alpha <2(\sqrt{2}-1)$ and β=1, then the WB PDF $z⟼f_{Z}\left(z;\alpha ,1\right)$ is decreasing.*
*2.*  *If $2(\sqrt{2}-1)=\alpha <1=\beta$, then the WB PDF $z⟼f_{Z}\left(z;\alpha ,1\right)$ has a U-shape, with minimum point z=α/[2(α+1)].*
*3.*  *If $2(\sqrt{2}-1)<\alpha <1=\beta$, then the WB PDF $z⟼f_{Z}\left(z;\alpha ,1\right)$ has is decreasing-increasing-decreasing (DID), with minimum point $z=\left[\alpha -\sqrt{\alpha^{2}+4\alpha -4}\;\right]/\left[2\left(\alpha +1\right)\right]$ and maximum point $z=\left[\alpha +\sqrt{\alpha^{2}+4\alpha -4}\;\right]/\left[2\left(\alpha +1\right)\right]$.*
*4.*  *If α>1=β, then the WB PDF $z⟼f_{Z}\left(z;\alpha ,1\right)$ is unimodal, with mode $z=\left[\alpha +\sqrt{\alpha^{2}+4\alpha -4}\;\right]/\left[2\left(\alpha +1\right)\right]$.*

Finally, when α≠1 and β≠1, upon taking the derivative of $f_{Z}\left(z;\alpha ,\beta \right)$ in (3), with respect to *z*, we get

(4) $$\begin{array}{c} \frac{df_{Z}\left(z;\alpha ,\beta \right)}{dz}=C\left(\alpha ,\beta \right)z^{\alpha -2}{\left(1-z\right)}^{\beta -2}p_{3}\left(z;\alpha ,\beta \right), \end{array}$$

where C(α,β)=(α+β+1)/[(1+α)(1+β)B(α,β)] and $p_{3}\left(z;\alpha ,\beta \right)$ is the cubic polynomial defined as

$$\begin{array}{c} p_{3}\left(z;\alpha ,\beta \right)={\left(\alpha +\beta \right)}^{2}z^{3}-\left(\alpha +\beta \right)\left(2\alpha +\beta \right)z^{2}+\left[\left(\alpha +\beta \right)\left(\alpha -1\right)+2\right]z+\alpha -1. \end{array}$$

By the Fundamental Theorem of Algebra, $p_{3}\left(z;\alpha ,\beta \right)$ has at least one and at most 3 real roots. In the next results, we analyze when any (or none) of these roots lie in (0,1). This lets us find the number of critical points (if any) of the WB PDF. Having found the critical points of the WB PDF, we can discover the shape of the WB PDF using the asymptotic behavior of the WB PDF.

**Theorem 1.** 
*The following hold:*

*1.*  *If α<1 and β<1, then the WB PDF $z\mapsto f_{Z}\left(z;\alpha ,\beta \right)$ is U-shaped or decreasing-increasing-decreasing-increasing.*
*2.*  *If α<1<β, then*
$$\left\{\begin{array}{cc} \mathrm{the}\;\mathrm{WB}\;\mathrm{PDF}\;\mathrm{is}\;\mathrm{decreasing}\text{-}\mathrm{increasing}\text{-}\mathrm{decreasing}, & \mathrm{if}\;\Delta (\alpha ,\beta )>0,\\ \mathrm{the}\;\mathrm{WB}\;\mathrm{PDF}\;\mathrm{is}\;\mathrm{decreasing}, & \mathrm{if}\;\Delta (\alpha ,\beta )<0,\\ \mathrm{the}\;\mathrm{WB}\;\mathrm{PDF}\;\mathrm{is}\;\mathrm{non}\text{-}\mathrm{increasing}, & \mathrm{if}\;\Delta (\alpha ,\beta )=0, \end{array}\right.$$
  *where Δ(α,β) denotes the discriminant of $p_{3}\left(z;\alpha ,\beta \right)$.*
*3.*  *If α>1>β, then*
$$\left\{\begin{array}{cc} \mathrm{the}\;\mathrm{WB}\;\mathrm{PDF}\;\mathrm{is}\;\mathrm{increasing}\text{-}\mathrm{decreasing}\text{-}\mathrm{increasing}, & \mathrm{if}\;\Delta (\alpha ,\beta )>0,\\ \mathrm{the}\;\mathrm{WB}\;\mathrm{PDF}\;\mathrm{is}\;\mathrm{non}\text{-}\mathrm{decreasing}, & \mathrm{if}\;\Delta (\alpha ,\beta )⩽0. \end{array}\right.$$
*4.*  *If α>1 and β>1, then the WB PDF $z\mapsto f_{Z}\left(z;\alpha ,\beta \right)$ is unimodal.*

The proof of Theorem 1 is reported in Appendix A.

## 3. Stochastic Properties of WB Model

In this section, we introduce different stochastic representations of the WB model. We also discuss two models in which the WB is a conjugate prior distribution and their corresponding posterior predictive distributions.

**Theorem 2** 
(Characterization–Stochastic representation)**.** *Let $Y_{1}$ and $Y_{2}$ be two independent random variables following the WL distribution (see [12]) with vector parameters (α,1) and (β,1), respectively, where α>0 and β>0 are shape parameters, i.e.,*

$$\begin{array}{cc} f_{Y_{1}}\left(x;\alpha \right)\; & =\frac{1}{\left(1+\alpha )\Gamma (\alpha \right)}\;x^{\alpha -1}\left(1+x\right)\exp\left(-x\right),\;x>0,\\ f_{Y_{2}}\left(y;\beta \right)\; & =\frac{1}{\left(1+\beta )\Gamma (\beta \right)}\;y^{\beta -1}\left(1+y\right)\exp\left(-y\right),\;y>0. \end{array}$$

*Then, the random variable has the stochastic representation*

$$Z\overset{d}{=}\frac{Y_{1}}{Y_{1}+Y_{2}}∼\mathrm{WB}\left(\alpha ,\beta \right),$$

*namely, Z is WB distributed with shape parameters α,β>0.*

The proof of Theorem 2 is omitted here because it is a straightforward consequence of Theorem 2 in [14]. For more details, see Example 8 in [14]. It should be noted that stochastic representations are important since they may justify some models arising naturally in real situations.

**Remark 1** 
([12])**.** *The WL distribution can be viewed as a mixture of two gamma distributions, namely, Gamma(α,β) and Gamma(α+1,β) with mixing proportions q=α/(α+β) and 1−q=β/(α+β), respectively, as*

$$f_{Y}\left(y;\alpha ,\beta \right)=\frac{\alpha }{\alpha +\beta }\mathrm{Gamma}\left(\alpha ,\beta \right)+\frac{\beta }{\alpha +\beta }\mathrm{Gamma}\left(\alpha +1,\beta \right),$$

*where the pdf of the Gamma distribution with parameters α and β is given by*

$$f_{G}\left(x;\alpha ,\beta \right)=\frac{\beta^{\alpha }x^{\alpha -1}}{\Gamma (\alpha )}e^{-\beta x}.$$

**Proposition 3** 
(Mixture)**.** *From (3), we have that*

$$\begin{array}{cc} f_{Z}\left(z;\alpha ,\beta \right)\; & =\frac{\left(\alpha +\beta +1\right)}{\left(1+\alpha )(1+\beta \right)}\;\frac{z^{\alpha -1}{\left(1-z\right)}^{\beta -1}}{B(\alpha ,\beta )}+\frac{\alpha \;\beta }{\left(1+\alpha )(1+\beta \right)}\;\frac{z^{\alpha }{\left(1-z\right)}^{\beta }}{B(\alpha +1,\beta +1)}\\ \; & =pf_{Z_{0}}\left(z\right)+\left(1-p\right)f_{Z_{1}}\left(z\right),\;0<z<1, \end{array}$$

*where p=(α+β+1)/[(α+1)(β+1)] and $Z_{j}∼\mathrm{Beta}\left(\alpha +j,\beta +j\right)$, j=0,1, i.e.,*

$$Z∼\left\{\begin{array}{cc} Z_{1},\;\; & \mathrm{with}\mathrm{probability}\;(\alpha +\beta +1)/[(\alpha +1)(\beta +1)],\\ Z_{2},\;\; & \mathrm{with}\mathrm{probability}\;(\alpha \;\beta )/[(\alpha +1)(\beta +1)], \end{array}\right.$$

*where $Z_{1}∼\mathrm{Beta}\left(\alpha ,\beta \right)$ and $Z_{2}∼\mathrm{Beta}\left(\alpha +1,\beta +1\right)$.*

Proposition 3 introduces a new class of finite mixtures of beta distributions that provides additional flexibility. This mixture of beta distributions has a certain advantage over competitors, in that it does not require a subjective approach involving guessing the mixing weights (known weights), i.e., the WB distribution can be viewed as a mixture of two beta distributions with known weights. The weights depend only on the parameters of the proposed distribution. For instance, ref. [15] proposed different kinds of beta-distribution mixtures. The beta mixture models proposed by [15] have four parameters. However, more flexibility comes at the cost of model complexity and additional parameters, making model specification and inference more difficult. Furthermore, the class of mixture of beta distributions proposed by [15] requires information in guessing the weights. For example, applications using finite mixtures from the beta distribution, see [16,17].

From Proposition 3 the cumulative distribution function (CDF) of the WB distribution is given by

(5) $$F_{Z}\left(z;\alpha ,\beta \right)=\frac{\left(\alpha +\beta +1\right)}{\left(1+\alpha )(1+\beta \right)}I_{x}\left(\alpha ,\beta \right)+\frac{\alpha \;\beta }{\left(1+\alpha )(1+\beta \right)}I_{x}\left(\alpha +1,\beta +1\right),$$

where $I_{x}\left(\alpha ,\beta \right)=B_{x}\left(\alpha ,\beta \right)/B\left(\alpha ,\beta \right)$ is the incomplete beta function ratio, $B_{x}\left(\alpha ,\beta \right)=\int_{0}^{x}t^{\alpha -1}{\left(1-t\right)}^{\beta -1}dt$ is the incomplete beta function.

The moments of the WB distribution are both immediate and obtainable from Remark 3

$$E\left(Z^{k}\right)=\frac{\left(\alpha +\beta +1\right)}{\left(1+\alpha )(1+\beta \right)}\prod_{r=0}^{k-1}\frac{\alpha +r}{\alpha +\beta +r}+\frac{\alpha \;\beta }{\left(1+\alpha )(1+\beta \right)}\prod_{r=0}^{k-1}\frac{\alpha +1+r}{\alpha +\beta +2+r}.$$

In particular, the mean and the variance are given by

$$E\left(Z\right)=\frac{\alpha (\alpha +\beta +1)}{\left(1+\alpha )(1+\beta )(\alpha +\beta \right)}+\frac{\alpha \;\beta }{\left(1+\beta )(\alpha +\beta +2\right)}$$

and

(6) $$\begin{array}{ccc} \mathrm{Var}(Z) & = & \frac{\alpha }{\left(1+\beta )(\alpha +\beta \right)}+\frac{\alpha \;\beta (\alpha +2)}{\left(1+\beta )(\alpha +\beta +2)(\alpha +\beta +3\right)}\\ & - & {\left[\frac{\alpha (\alpha +\beta +1)}{\left(1+\alpha )(1+\beta )(\alpha +\beta \right)}+\frac{\alpha \;\beta }{\left(1+\beta )(\alpha +\beta +2\right)}\right]}^{2}. \end{array}$$

**Corollary 1.** 
*From Proposition 3 and Equation (1),*

$$Z=p\frac{G_{0}}{G_{0}+W_{0}}+\left(1-p\right)\frac{G_{1}}{G_{1}+W_{1}},$$

*where p=(α+β+1)/[(α+1)(β+1)], $G_{j}∼\mathrm{Gamma}\left(\alpha +j,\theta \right)$ and $W_{j}∼\mathrm{Gamma}\left(\beta +j,\theta \right)$ are independent, j=0,1. Therefore, the WB distribution can also be written as a function of gamma-distributed r.v.s.*

**Corollary 2.** 
*Based on Corollary 1, we also can establish that*

$$\begin{array}{c} Z|(U=u)∼\mathrm{Beta}\left(\alpha +u,\beta +u\right)\;and\;U∼\mathrm{Bernoulli}\left(\frac{\alpha \beta }{\left(\alpha +1)(\beta +1\right)}\right),\;u=0,1, \end{array}$$

*where U∈{0,1} is independent of $G_{j}/\left(G_{j}+W_{j}\right)$, j = *0, 1*. In other words, the WB distribution can be written as a Beta distribution, where a Bernoulli r.v. is added to both shape parameters. Thus, the WB distribution is a type of discrete mixed Beta distribution [13].*

According to [13], the mixing distributions are certain discrete distributions with a parameter that is itself beta distributed.

**Remark 2.** 
*Let B be a variable following the beta distribution defined in (1). It is easy to see that*

$$\begin{array}{c} B|\left(U=u\right)∼\mathrm{Beta}\left(\alpha +u,\beta +\left(1-u\right)\right)\;\mathrm{and}\;U∼\mathrm{Bernoulli}\left(\frac{\alpha }{\alpha +\beta }\right),\;u=0,1, \end{array}$$

*that is,*

$$\begin{array}{c} X=UB_{1}+\left(1-U\right)B_{2}, \end{array}$$

*where U is independent of $B_{1}∼\mathrm{Beta}\left(\alpha +1,\beta \right)$ and $B_{2}∼\mathrm{Beta}\left(\alpha ,\beta +1\right)$.*

**Proposition 4.** 
*Let Z be a variable following the WB distribution defined in (3). The following results are valid for the WB distribution:*

*1.*  *The random variable W=Z/[Z+λ(1−Z)] has weighted Libby-Novick distribution (new distribution) with parameters α,β,λ>0, and density*
$$\begin{array}{ccc} f_{W}\left(w\right) & = & \frac{\lambda^{\alpha }\left(\alpha +\beta +1\right)}{\left(1+\alpha )(1+\beta \right)}\;\frac{w^{\alpha -1}{\left(1-w\right)}^{\beta -1}}{B\left(\alpha ,\beta \right){\left[1-\left(1-\lambda \right)w\right]}^{\alpha +\beta }}\left\{\left(\alpha +\beta \right)\;\frac{\lambda w(1-w)}{{\left(1-\left(1-\lambda \right)w\right)}^{2}}+1\right\}\\ & = & pf_{W_{0}}\left(z\right)+\left(1-p\right)f_{W_{1}}\left(z\right),\;0<w<1, \end{array}$$
  *where p=(α+β+1)/[(α+1)(β+1)], $W_{j}∼\mathrm{Libby}-\mathrm{Novick}\left(\alpha +j,\beta +j,\lambda \right)$ [18], j=0,1.*
*2.*  *The random variable V=Z/(1−Z) has a weighted beta prime distribution (new distribution) with parameters α,β>0, and density*
(7) $$\begin{array}{ccc} f_{V}\left(v;\alpha ,\beta \right) & = & \frac{\left(\alpha +\beta +1\right)}{\left(1+\alpha )(1+\beta \right)}\;\frac{v^{\alpha -1}{\left(1+v\right)}^{-\alpha -\beta }}{B(\alpha ,\beta )}\left[\frac{(\alpha +\beta )v}{{\left(1+v\right)}^{2}}+1\right]\\ & = & pf_{V_{0}}\left(v\right)+\left(1-p\right)f_{V_{1}}\left(v\right),\;v>0, \end{array}$$
  *where p=(α+β+1)/[(α+1)(β+1)], $V_{j}∼\mathrm{Beta}\;\mathrm{prime}\left(\alpha +j,\beta +j\right)$ [1], j=0,1.*

**Proposition 5.** 
*Let $Y_{1}$ and $Y_{2}$ be two independent random variables following standard WL distributions (scale parameter equal to 1) with shape parameters α>0 and β>0. Then, the random variable V has the stochastic representation*

$$V\overset{d}{=}\frac{Y_{1}}{Y_{2}}∼\mathrm{WBP}\left(\alpha ,\beta \right),$$

*namely, V is weighted beta prime (WBP) distributed, defined in (7) with shape parameters α,β>0.*

The proof of Proposition 5 is omitted here since it is a straightforward consequence of Theorem 2 above.

**Proposition 6.** 
*Let Z be a variable following the WB distribution defined in (3).*

*1.*  *Consider M|Z=z∼Binomial(n,z), then the marginal distribution of M is a mixture beta-binomial distribution (new distribution) with parameters n∈N, α,β>0, and probability mass function*
(8) $$\begin{array}{ccc} P(M=k) & = & \frac{\left(\alpha +\beta +1\right)}{\left(1+\alpha )(1+\beta \right)}\;\frac{B(k+\alpha ,n-k+\beta )}{B(\alpha ,\beta )}\\ & + & \frac{\alpha \beta }{\left(1+\alpha )(1+\beta \right)}\;\frac{B(k+\alpha +1,n-k+\beta +1)}{B(\alpha +1,\beta +1)}\\ & = & pP\left(M_{0}=k\right)+\left(1-p\right)P\left(M_{1}=k\right),\;k=0,1,\ldots ,n, \end{array}$$
  *where p=(α+β+1)/[(α+1)(β+1)] and $M_{j}∼\mathrm{Beta}-\mathrm{Binomial}\left(n,\alpha +j,\beta +j\right)$, j=0,1.*
*2.*  *Consider M|Z=z∼Negativebinomial(r,z), then the marginal distribution of M is a mixture beta-negative binomial distribution (new distribution) with parameters r,α,β>0, and probability mass function*
(9) $$\begin{array}{ccc} P(M=k)= & = & \frac{\left(\alpha +\beta +1\right)}{\left(1+\alpha )(1+\beta \right)}\;\frac{B(r+k,\alpha +\beta )}{B(r,\alpha )}\frac{\Gamma (k+\beta )}{k!\Gamma (\beta )}\\ & + & \frac{\alpha \beta }{\left(1+\alpha )(1+\beta \right)}\frac{B(r+k,\alpha +\beta +2)}{B(r,\alpha +1)}\frac{\Gamma (k+\beta +1)}{k!\Gamma (\beta +1)}\;\\ & = & pP\left(M_{0}=k\right)+\left(1-p\right)P\left(M_{1}=k\right),\;k=0,1,2,\ldots , \end{array}$$
  *where*
$$P\left(M=k|Z=z\right)=\frac{\Gamma (r+k)}{\Gamma (r)k!}z^{r}{\left(1-z\right)}^{k},\;k=0,1,2,\ldots ,$$
*p=(α+β+1)/[(α+1)(β+1)] and $M_{j}∼\mathrm{Beta}-\mathrm{negative}\;\mathrm{binomial}\left(r,\alpha +j,\beta +j\right)$, j=0,1.*

We can use the distributions proposed in Proposition 6 as posterior predictive distributions. In Bayesian statistics, the posterior predictive distribution is the distribution of unobserved observations (prediction) conditional on the observed data. When the conditional distribution of the observation M|Z=z is binomial (n,z), with Z∼WB(α,β), the posterior predictive distribution is given by (8). On the other hand, when the conditional distribution of the observation M|Z=z is negative binomial (r,z) with Z∼WB(α,β), the posterior predictive distribution is given by (9).

## 4. Regression Analysis and Inference

In this section, we discuss a regression model for the WB model. Let $Z_{1},\ldots ,Z_{n}$ be *n* independent random variables, where each $Z_{i}$, i=1,…,n, follows the PDF given in (3). We assume that the parameters $\alpha_{i}$ and $\beta_{i}$ satisfy the following functional relations:

(10) $$g_{1}\left(\alpha_{i}\right)=\eta_{1i}=w_{i}^{⊤}\gamma \;\mathrm{and}\;g_{2}\left(\beta_{i}\right)=\eta_{2i}=y_{i}^{⊤}\zeta ,$$

where $\gamma ={\left(\gamma_{1},\ldots ,\gamma_{k}\right)}^{⊤}$ and $\zeta ={\left(\zeta_{1},\ldots ,\zeta_{l}\right)}^{⊤}$ are vectors of unknown regression coefficients which are assumed to be functionally independent, $\gamma \in R^{k}$ and $\zeta \in R^{l}$, with k+l<n, $\eta_{1i}$ and $\eta_{2i}$ are the linear predictors, and $w_{i}={\left(w_{i1},\ldots ,w_{ik}\right)}^{⊤}$ and $y_{i}={\left(y_{i1},\ldots ,y_{il}\right)}^{⊤}$ are observations on *k* and *l* known regressors, for i=1,…,n. Furthermore, we assume that the covariate matrices $W={\left(w_{1},\ldots ,w_{n}\right)}^{⊤}$ and $Y={\left(y_{1},\ldots ,y_{n}\right)}^{⊤}$ have rank *k* and *l*, respectively. The link functions $g_{1}:R\to R^{+}$ and $g_{2}:R\to R^{+}$ in (10) must be strictly monotone, positive and at least twice differentiable, such that $\alpha_{i}=g_{1}^{-1}\left(x_{i}^{⊤}\;\gamma \right)$ and $\beta_{i}=g_{2}^{-1}\left(y_{i}^{⊤}\;\zeta \right)$, with $g_{1}^{-1}\left(\cdot \right)$ and $g_{2}^{-1}\left(\cdot \right)$ being the inverse functions of $g_{1}\left(\cdot \right)$ and $g_{2}\left(\cdot \right)$, respectively.

**Remark 3.** 
*From Remark 1, the WL(α,1) model can be written as a mixture of Gamma$\left(\alpha_{i},1\right)$ and Gamma$\left(\alpha_{i}+1,1\right)$ distributions, where the mixing probability is given by $q_{i}=\alpha_{i}/\left(1+\alpha_{i}\right)$, or equivalently, $\mathrm{logit}\left(q_{i}\right)=\log\left(\alpha_{i}\right)$, where logit(q)=log(q/(1−q)) denotes the logarithm of the odds ratio. Therefore,*

$$\mathrm{logit}\left(q_{i}\right)=w_{i}^{⊤}\gamma .$$

*Suppose that the j-th covariate is quantitative. Let $w_{i}^{⋆}$ be the vector $w_{i}$, but with the j-th component increased in one unit and $q_{i}^{⋆}$ the corresponding mixing probability related to $w_{i}^{⋆}$. Note that $w_{i}^{⋆⊤}\gamma =w_{i}^{⊤}\gamma +\gamma_{j}$. Then,*

$$\mathrm{logit}\left(q_{i}\right)-\mathrm{logit}\left(q_{i}^{⋆}\right)=\gamma_{j}.$$

*Therefore, $\gamma_{j}$ can be interpreted as the increment (or decrement) in the logarithm of the odds ratio for the mixing probability in $Y_{1}$. Similarly, we can show that $\zeta_{j}$ represents the increment (or decrement) in the logarithm of the odds ratio for the mixing probability in $Y_{2}$. A similar interpretation holds when the j-th covariate is dichotomic.*

Furthermore, as established in Section 2, the WB distribution also admits a finite mixture representation $Z_{i}∼p_{i}\mathrm{Beta}\left(\alpha_{i},\beta_{i}\right)+\left(1-p_{i}\right)\mathrm{Beta}\left(\alpha_{i}+1,\beta_{i}+1\right)$, where the mixing weight $p_{i}=\frac{\alpha_{i}+\beta_{i}+1}{\left(\alpha_{i}+1\right)\left(\beta_{i}+1\right)}$ is explicitly controlled by $\alpha_{i}$ and $\beta_{i}$. Consequently, linking covariates to $\alpha_{i}$ and $\beta_{i}$ through $g_{1}\left(\alpha_{i}\right)=w_{i}^{⊤}\gamma$ and $g_{2}\left(\beta_{i}\right)=y_{i}^{⊤}\zeta$ directly governs the allocation weight $p_{i}$ between the two Beta component distributions.

Therefore, although the proposed regression model is not directly indexed by the conditional mean, its regression coefficients have a natural interpretation through both the stochastic Lindley representation and the underlying Beta mixture structure. This formulation is fully consistent with modern distributional regression frameworks (such as GAMLSS), where covariates may be associated with parameters governing shape, skewness, kurtosis, and tail behaviors, rather than exclusively with the conditional mean.

Let $\theta ={\left(\gamma^{⊤},\zeta^{⊤}\right)}^{⊤}$ be the parameter vector associated with the WB regression model. The log-likelihood function of θ, given the observed sample $Y={\left(y_{1},y_{2},\ldots ,y_{n}\right)}^{⊤}$ has the form

(11) $$\begin{array}{c} \ell \left(\gamma ,\zeta \right)=\sum_{i=1}^{n}\ell \left(\alpha_{i},\beta_{i}\right), \end{array}$$

where

(12) $$\begin{array}{ccc} \ell (\alpha_{i},\beta_{i}) & = & \log\left(\alpha_{i}+\beta_{i}+1\right)-\log\left(1+\alpha_{i}\right)-\log\left(1+\beta_{i}\right)-\log\left[B\left(\alpha_{i},\beta_{i}\right)\right]\\ & + & \left(\alpha_{i}-1\right)\log\left(z_{i}\right)+\left(\beta_{i}-1\right)\log\left(1-z_{i}\right)+\log\left[\left(\alpha_{i}+\beta_{i}\right)z_{i}\left(1-z_{i}\right)+1\right]. \end{array}$$

The score vector, obtained by differentiating the log-likelihood function, given in (11), with respect to the γ and ζ has components

$$\begin{array}{cc} \frac{\partial \ell (\gamma ,\zeta )}{\partial \gamma_{r}}\; & =\sum_{i=1}^{n}\frac{\partial \ell (\alpha_{i},\beta_{i})}{\partial \alpha_{i}}\;\frac{\partial \alpha_{i}}{\partial \gamma_{r}},\;r=1,\ldots ,k,\\ \frac{\partial \ell (\gamma ,\zeta )}{\partial \zeta_{s}}\; & =\sum_{i=1}^{n}\frac{\partial \ell (\alpha_{i},\beta_{i})}{\partial \beta_{i}}\;\frac{\partial \beta_{i}}{\partial \zeta_{s}},\;s=1,\ldots ,l, \end{array}$$

where (for i=1,…,n)

(13) $$\begin{array}{cc} \frac{\partial \ell (\alpha_{i},\beta_{i})}{\partial \alpha_{i}}\; & =\frac{1}{\alpha_{i}+\beta_{i}+1}-\frac{1}{\alpha_{i}+1}-\left[\psi^{\left(0\right)}\left(\alpha_{i}\right)-\psi^{\left(0\right)}\left(\alpha_{i}+\beta_{i}\right)\right]+\log\left(z_{i}\right)+\frac{z_{i}\left(1-z_{i}\right)}{\left(\alpha_{i}+\beta_{i}\right)z_{i}\left(1-z_{i}\right)+1}, \end{array}$$

(14) $$\begin{array}{cc} \frac{\partial \ell (\alpha_{i},\beta_{i})}{\partial \beta_{i}}\; & =\frac{1}{\alpha_{i}+\beta_{i}+1}-\frac{1}{\beta_{i}+1}-\left[\psi^{\left(0\right)}\left(\beta_{i}\right)-\psi^{\left(0\right)}\left(\alpha_{i}+\beta_{i}\right)\right]+\log\left(1-z_{i}\right)+\frac{z_{i}\left(1-z_{i}\right)}{\left(\alpha_{i}+\beta_{i}\right)z_{i}\left(1-z_{i}\right)+1}, \end{array}$$

and

(15) $$\begin{array}{c} \frac{\partial \alpha_{i}}{\partial \gamma_{r}}=\frac{w_{ir}}{g_{1}^{\prime }\left(\alpha_{i}\right)},\;r=1,\ldots ,k, \end{array}$$

(16) $$\begin{array}{c} \frac{\partial \beta_{i}}{\partial \zeta_{s}}=\frac{y_{is}}{g_{2}^{\prime }\left(\beta_{i}\right)},\;s=1,\ldots ,l \end{array}$$

with $\psi^{\left(n\right)}\left(x\right)$ is the *n*th derivative of the digamma function.

The ML estimator of θ is the value $\hat{\theta }={\left({\hat{\gamma }}^{⊤},{\hat{\zeta }}^{⊤}\right)}^{⊤}$ that maximizes ℓ(γ,ζ). Because setting the first-order log-likelihood derivatives to zero yields a complicated system of nonlinear equations, we compute the maximum likelihood estimate of θ using numerical methods. We performed this numerical maximization using the RS and CG algorithms described by [19] and implemented in R (Version 4.4.1, available at http://www.r-project.org) via the gamlss package.

The observed information matrix at $\left(\gamma^{*},\zeta^{*}\right)$ is given by

$$\begin{array}{c} J\left(\gamma^{*},\zeta^{*}\right)=-\left[\frac{\partial^{2}}{\partial \theta \partial \theta^{\prime }}\right]\;\ell \left(\gamma ,\zeta \right)\;{|}_{\left(\gamma ,\zeta \right)=\left(\gamma^{*},\zeta^{*}\right)}, \end{array}$$

where $\theta ,\theta^{\prime }\in \left\{\gamma_{1},\ldots ,\gamma_{k},\zeta_{1},\ldots ,\zeta_{l}\right\}$,

$$\begin{array}{cc} \frac{\partial^{2}\ell \left(\gamma ,\zeta \right)}{\partial \gamma_{r}^{2}}\; & =\sum_{i=1}^{n}\left[\frac{\partial^{2}\ell \left(\alpha_{i},\beta_{i}\right)}{\partial \alpha_{i}^{2}}\;{\left(\frac{\partial \alpha_{i}}{\partial \gamma_{r}}\right)}^{2}+\frac{\partial \ell (\alpha_{i},\beta_{i})}{\partial \alpha_{i}}\;\frac{\partial^{2}\alpha_{i}}{\partial \gamma_{r}^{2}}\right],\;r=1,\ldots ,k,\\ \frac{\partial \ell (\gamma ,\zeta )}{\partial \zeta_{s}}\; & =\sum_{i=1}^{n}\left[\frac{\partial^{2}\ell \left(\alpha_{i},\beta_{i}\right)}{\partial \beta_{i}^{2}}\;{\left(\frac{\partial \beta_{i}}{\partial \zeta_{s}}\right)}^{2}+\frac{\partial \ell (\alpha_{i},\beta_{i})}{\partial \beta_{i}}\;\frac{\partial^{2}\beta_{i}}{\partial \zeta_{s}^{2}}\right],\;s=1,\ldots ,l,\\ \frac{\partial^{2}\ell \left(\gamma ,\zeta \right)}{\partial \zeta_{s}\partial \gamma_{r}}\; & =\sum_{i=1}^{n}\left[\frac{\partial^{2}\ell \left(\alpha_{i},\beta_{i}\right)}{\partial \beta_{i}\alpha_{i}}\;\frac{\partial \beta_{i}}{\partial \zeta_{s}}\;\frac{\partial \alpha_{i}}{\partial \gamma_{r}}+\frac{\partial \ell (\alpha_{i},\beta_{i})}{\partial \alpha_{i}}\;\frac{\partial^{2}\alpha_{i}}{\partial \zeta_{s}\gamma_{r}}\right],\;r=1,\ldots ,k,\;s=1,\ldots ,l, \end{array}$$

and (for i=1,…,n)

$$\begin{array}{cc} \frac{\partial^{2}\ell \left(\alpha_{i},\beta_{i}\right)}{\partial \alpha_{i}^{2}}\; & =-\frac{1}{{\left(\alpha_{i}+\beta_{i}+1\right)}^{2}}+\frac{1}{{\left(\alpha_{i}+1\right)}^{2}}-\left[\psi^{\left(1\right)}\left(\alpha_{i}\right)-\psi^{\left(1\right)}\left(\alpha_{i}+\beta_{i}\right)\right]-\frac{z_{i}^{2}{\left(1-z_{i}\right)}^{2}}{{\left[\left(\alpha_{i}+\beta_{i}\right)z_{i}\left(1-z_{i}\right)+1\right]}^{2}},\\ \frac{\partial^{2}\ell \left(\alpha_{i},\beta_{i}\right)}{\partial \beta_{i}^{2}}\; & =-\frac{1}{{\left(\alpha_{i}+\beta_{i}+1\right)}^{2}}+\frac{1}{{\left(\beta_{i}+1\right)}^{2}}-\left[\psi^{\left(1\right)}\left(\beta_{i}\right)-\psi^{\left(1\right)}\left(\alpha_{i}+\beta_{i}\right)\right]+\frac{z_{i}^{2}{\left(1-z_{i}\right)}^{2}}{{\left[\left(\alpha_{i}+\beta_{i}\right)z_{i}\left(1-z_{i}\right)+1\right]}^{2}},\\ \frac{\partial^{2}\ell \left(\alpha_{i},\beta_{i}\right)}{\partial \beta_{i}\alpha_{i}}\; & =-\frac{1}{{\left(\alpha_{i}+\beta_{i}+1\right)}^{2}}+\psi^{\left(1\right)}\left(\alpha_{i}+\beta_{i}\right)+\frac{z_{i}^{2}{\left(1-z_{i}\right)}^{2}}{{\left[\left(\alpha_{i}+\beta_{i}\right)z_{i}\left(1-z_{i}\right)+1\right]}^{2}}, \end{array}$$

with

$$\begin{array}{cc} \frac{\partial^{2}\alpha_{i}}{\partial \gamma_{r}^{2}} & =-\frac{g_{1}^{\prime \prime }\left(\alpha_{i}\right)}{{\left[g_{1}^{\prime }\left(\alpha_{i}\right)\right]}^{3}}w_{ir}^{2},\;r=1,\ldots ,k,\\ \frac{\partial^{2}\beta_{i}}{\partial \zeta_{s}^{2}} & =-\frac{g_{2}^{\prime \prime }\left(\beta_{i}\right)}{{\left[g_{2}^{\prime }\left(\beta_{i}\right)\right]}^{3}}y_{is}^{2},\;s=1,\ldots ,l,\\ \frac{\partial^{2}\alpha_{i}}{\partial \gamma_{r}\partial \gamma_{s}} & =-\frac{g_{1}^{\prime \prime }\left(\alpha_{i}\right)}{{\left[g_{1}^{\prime }\left(\alpha_{i}\right)\right]}^{3}}w_{ir}w_{is},\;r=1,\ldots ,k,\;s=1,\ldots ,l.\\ \frac{\partial^{2}\beta_{i}}{\partial \zeta_{s}\partial \zeta_{t}} & =-\frac{g_{2}^{\prime \prime }\left(\beta_{i}\right)}{{\left[g_{2}^{\prime }\left(\beta_{i}\right)\right]}^{3}}y_{is}y_{it},\;r=1,\ldots ,k,\;s=1,\ldots ,l.\\ \frac{\partial^{2}\alpha_{i}}{\partial \zeta_{s}\gamma_{r}}\; & =0,\;r=1,\ldots ,k,\;s=1,\ldots ,l. \end{array}$$

Under usual regularity conditions (see [20]), for a large sample size, it follows that the maximum likelihood estimator θ has (k+l)-variate normal asymptotic distribution with mean θ and variance $K{\left(\theta \right)}^{-1}$, denoted by

$$\hat{\theta }=\left(\begin{array}{c} \hat{\gamma }\\ \hat{\zeta } \end{array}\right)\overset{a}{∼}N_{k+l}\left(\theta ,K{\left(\theta \right)}^{-1}\right),$$

where $\overset{a}{∼}$ means “asymptotically distributed according to the distribution,” or “has asymptotic distribution,” and K(θ) is the Fisher information matrix. Unfortunately, K(θ) has no closed-form expression. Nevertheless, a consistent estimator of the expected Fisher information matrix is given by

$$J\left(\hat{\theta }\right)=-\;\frac{\partial \ell \left(\theta \right)}{\partial \theta \;\partial \theta^{⊤}}{|}_{\theta =\hat{\theta }}\;,$$

which is the estimated observed Fisher information matrix. Therefore, for large *n*, we can approximate K(θ) by $J(\hat{\theta })$ (see Table 1).

## 5. Diagnostic

In this section, we discuss local influence analysis for the WB regression model and analyze its residuals using randomized quantile residuals.

### 5.1. Residual Analysis

In order to evaluate and study departures from the model assumption, we propose to use the quantile residuals (QR) introduced by [21]. Consider the CDF F(z;α,β) of the WB distribution given in (5). Since F(·) is continuous, then F(Z;α,β)∼U(0,1). Then, the quantile residual [21] under the WB regression model is defined by

$$r_{i}^{Q}=\Phi^{-1}\left\{F\left(z_{i};{\hat{\alpha }}_{i},{\hat{\beta }}_{i}\right)\right\},\;i=1,2,\ldots ,n,$$

where Φ(·) is the cumulative distribution function of the standard normal distribution, ${\hat{\alpha }}_{i}=g_{1}^{-1}\left(x_{i}^{⊤}\;\hat{\gamma }\right)$ and ${\hat{\beta }}_{i}=g_{2}^{-1}\left(y_{i}^{⊤}\;\hat{\zeta }\right)$. Except for variability due to parameter estimation, the distribution of the QR is standard normal.

We can perform formal goodness-of-fit tests in order to verify the distribution of the QR. Ref. [22] proposed a general approximate goodness-of-fit test for the hypothesis $H_{0},r_{1}^{Q},\ldots ,r_{n}^{Q}$ with $r_{i}^{Q}$ following F(x;θ), i.e., under $H_{0},r_{1}^{Q},\ldots ,r_{n}^{Q}$ is a random sample from a continuous distribution with cumulative distribution F(x;θ), where the form of *F* is known but the (k+l)-vector θ is unknown. The method is based on the Cramér–von Mises (CVM) and Anderson–Darling (AD) statistics. Furthermore, to test this hypothesis, we can also use the Kolmogorov–Smirnov (KS) and Shapiro–Wilk (SW) tests.

### 5.2. Local Influence

Local influence analysis is based on the curvature of the log-likelihood decision surface. Consider the WB regression model given in (10), with $\theta ={\left(\gamma^{⊤},\zeta^{⊤}\right)}^{⊤}$ being the parameter vector of unknown regression coefficients and ℓ(θ,ω) the log-likelihood function corresponding to this model defined in (12), but perturbed by a vector of perturbations ω.

The normal curvature for $\hat{\theta }$ in the arbitrary direction l, with ∥l∥=1, is given by $C_{l}\left(\hat{\theta }\right)=2\left|l^{⊤}\Delta^{⊤}{\hat{J}}^{-1}\Delta l\right|$, where $\hat{J}$ is the Hessian matrix of the log-likelihood function evaluated at $\hat{\theta }$, and Δ is a (k+l)×n perturbation matrix with elements

$$\Delta_{ij}={\left.\frac{\partial^{2}\ell \left(\theta ,\omega \right)}{\partial \theta_{i}\partial \omega_{j}}\right|}_{\theta =\hat{\theta },\;\omega =\omega_{0}},\;i=1,\ldots ,k+l,\;j=1,\ldots ,n,$$

evaluated at the non-perturbation vector $\omega_{0}$. The maximization of $C_{l}\left(\hat{\theta }\right)$ is equivalent to finding the largest eigenvalue $C_{\max}$ of the matrix $\hat{B}=\Delta^{⊤}{\hat{J}}^{-1}\Delta$, and the direction of largest perturbation around $\omega_{0}$, denoted by $l_{\max}$, is the corresponding eigenvector. The index plot of $l_{\max}$ helps assess the influence of small perturbations on the likelihood displacement. In what follows, we discuss two perturbation schemes: case-weight perturbation and response perturbation.

#### 5.2.1. Case-Weight Perturbation

Let $\omega ={\left(\omega_{1},\ldots ,\omega_{n}\right)}^{⊤}$ be a vector of case weights. Under this scheme, the perturbed log-likelihood function is given by $\ell \left(\theta ,\omega \right)=\sum_{j=1}^{n}\omega_{j}\ell \left(\alpha_{j},\beta_{j}\right)$, where $\ell (\alpha_{j},\beta_{j})$ is defined in (12), with $0\leq \omega_{j}\leq 1$, for j=1,…,n, and $\omega_{0}=1$ (the all-one vector). Thus, the elements of the perturbation matrix simplify to (for j=1,…,n):

$$\Delta_{ij}=\frac{\partial \ell (\alpha_{j},\beta_{j})}{\partial \vartheta }\frac{\partial \vartheta }{\partial \theta_{i}},\;\vartheta \in \left\{\alpha_{j},\beta_{j}\right\},\;\theta_{i}\in \left\{\gamma_{1},\ldots ,\gamma_{k},\zeta_{1},\ldots ,\zeta_{l}\right\},$$

where $\partial \ell (\alpha_{j},\beta_{j})/\partial \vartheta$ and $\partial \vartheta /\partial \theta_{i}$ are given in (13)–(14) and (15)–(16), respectively.

#### 5.2.2. Response Perturbation

Consider an additive perturbation on the *j*th response given by $z_{j}\left(\omega_{j}\right)=z_{j}+\omega_{j}s\left(z_{j}\right)$, where $s\left(z_{j}\right)=\sqrt{\mathrm{Var}(Z_{j})}$ is defined as in (6) and $\omega_{j}\in R$, for j=1,…,n. Under response perturbation, the log-likelihood function is $\ell \left(\theta ,\omega \right)=\sum_{j=1}^{n}\ell \left(\alpha_{j},\beta_{j};\omega_{j}\right)$, where

$$\begin{array}{cc} \ell (\alpha_{j},\beta_{j};\omega_{j}) & =\log\left(\alpha_{j}+\beta_{j}+1\right)-\log\left(1+\alpha_{j}\right)-\log\left(1+\beta_{j}\right)-\log\left[B\left(\alpha_{j},\beta_{j}\right)\right]\\ \; & \;+\left(\alpha_{j}-1\right)\log\left(z_{j}\left(\omega_{j}\right)\right)+\left(\beta_{j}-1\right)\log\left(1-z_{j}\left(\omega_{j}\right)\right)\\ \; & \;+\log[\left(\alpha_{j}+\beta_{j}\right)z_{j}\left(\omega_{j}\right)\left(1-z_{j}\left(\omega_{j}\right)\right)+1], \end{array}$$

with $\alpha_{j}=g_{1}^{-1}\left(w_{j}^{⊤}\gamma \right)$, $\beta_{j}=g_{2}^{-1}\left(y_{j}^{⊤}\zeta \right)$, and $\omega_{0}=0$ (null vector). Applying the chain and product rules, the elements of the perturbation matrix evaluated at $\omega_{0}=0$ take the form (for j=1,…,n):

$$\Delta_{ij}=\left[\frac{\partial^{2}\ell \left(\alpha_{j},\beta_{j}\right)}{\partial \vartheta \partial z_{j}}s\left(z_{j}\right)+\frac{\partial \ell (\alpha_{j},\beta_{j})}{\partial z_{j}}\frac{\partial s(z_{j})}{\partial \vartheta }\right]\frac{\partial \vartheta }{\partial \theta_{i}},$$

for $\vartheta \in \{\alpha_{j},\beta_{j}\}$ and $\theta_{i}\in \left\{\gamma_{1},\ldots ,\gamma_{k},\zeta_{1},\ldots ,\zeta_{l}\right\}$, where

$$\begin{array}{cc} \frac{\partial \ell (\alpha_{j},\beta_{j})}{\partial z_{j}} & =\frac{\alpha_{j}-1}{z_{j}}-\frac{\beta_{j}-1}{1-z_{j}}+\frac{\left(\alpha_{j}+\beta_{j}\right)\left(1-2z_{j}\right)}{\left(\alpha_{j}+\beta_{j}\right)z_{j}\left(1-z_{j}\right)+1},\\ \frac{\partial^{2}\ell \left(\alpha_{j},\beta_{j}\right)}{\partial \alpha_{j}\partial z_{j}} & =\frac{1}{z_{j}}+\frac{1-2z_{j}}{{\left[\left(\alpha_{j}+\beta_{j}\right)z_{j}\left(1-z_{j}\right)+1\right]}^{2}},\\ \frac{\partial^{2}\ell \left(\alpha_{j},\beta_{j}\right)}{\partial \beta_{j}\partial z_{j}} & =-\frac{1}{1-z_{j}}+\frac{1-2z_{j}}{{\left[\left(\alpha_{j}+\beta_{j}\right)z_{j}\left(1-z_{j}\right)+1\right]}^{2}}, \end{array}$$

and $\frac{\partial s(z_{j})}{\partial \vartheta }=\frac{1}{2\sqrt{\mathrm{Var}(Z_{j})}}\frac{\partial \mathrm{Var}(Z_{j})}{\partial \vartheta }$, with $\mathrm{Var}(Z_{j})$ and $\frac{\partial \vartheta }{\partial \theta_{i}}$ given in (6) and (15)–(16), respectively.

## 6. Simulation Study

In this section, we present two simulation studies. The Monte Carlo simulation experiments were performed using the R environment; see http://www.r-project.org. The first study evaluates the performance of the maximum likelihood estimators obtained within the GAMLSS framework for the WB regression model. The second evaluates the effectiveness of the quantile residuals for the WB regression model, also computed within the GAMLSS framework. The WB random variables are generated directly using the mixture representation given in Proposition 3, as follows:

1. Generate U∼Uniform(0,1);
2. If
  $$U\leq p=\frac{\alpha +\beta +1}{\left(\alpha +1)(\beta +1\right)},$$
  generate Z∼Beta(α,β);
3. Otherwise, generate Z∼Beta(α+1,β+1).

### 6.1. Parameter Recovery

In this subsection, we study the performance of the MLE for the WB regression model obtained via gamlss. For the parameters, we consider two scenarios:

- Case I: γ=(0.8,−0.3,0.3,0.4) and ζ=(−0.2,−0.3,0.1,0.1).
- Case II: γ=(−1.0,0.5,−0.2,0.6) and ζ=(0.5,−0.2,0.1,−0.4).

We also consider four sample sizes: 50, 100, 200 and 500. For each parameter combination and sample size, we draw 1000 replicates of the corresponding sample size and compute the estimators for each replicate. For each estimator, we summarize the average bias (bias), the average estimated standard error (SE), the root of the estimated mean squared error (RMSE), and the 95% coverage probabilities (CP). Table 1 presents the results. As the sample size increases, bias and SE decrease, and SE and RMSE become closer. Moreover, the CP terms approach the nominal value as the sample size increases. However, based on the results, we suggest caution when using the model with very small sample sizes (say n<<100) because the asymptotic distribution of the estimators is poor and can lead to inferential errors.

### 6.2. Assessing the Quantile Residuals

In this subsection, we discuss the validity of the QR discussed in Section 5.1. Such residuals are very simple to compute in a gamlss framework. Hence, we need to study its validity. For this, we use the same values for γ and ζ as in the previous study (cases I and II). However, in this case, the data were simulated not only from the WB model but also from the beta model. In both cases, we estimated both models and computed the QR. For both models, we checked the normality of the QR using the *p*-values from the KS, SW, AD, and CVM tests. We made decisions at the 5% significance level. Table 2 shows the percentage of times the respective test does not reject the normality hypothesis for the QR and the percentage of times each model provides a higher log-likelihood. The first conclusion is that the KS test is not appropriate for assessing the normality assumption for the QR because it is not practically rejected in any case. The SW, AD, and CVM tests do not reject the normality assumption for the correct model in virtually 95% of cases. However, the tests also do not reject such assumption in a high percentage of times for the model that is not the true, but as *n* increases, that percentage decreases. Similar conclusions hold for the log-likelihood function.

## 7. Real-World Data Analysis

In this section, we apply the proposed regression model to real data for illustration. We implemented the required numerical evaluations for data analysis in R. The dataset is included in the betareg package and contains responses from naive mock jurors to a conventional two-option verdict (guilt vs. acquittal) versus a three-option verdict setup (the third option was the Scottish ’not proven’ alternative), with or without conflicting testimonial evidence. The dataset contains 104 observations. The main objective of the analysis is to explain the variable *confidence*, which represents the juror’s degree of confidence in his/her verdict, scaled to the open unit interval, in terms of *verdict*, a factor indicating whether a two-option or three-option verdict is requested, and *conflict*, a factor indicating the presence or absence of conflicting testimonial evidence. We compared the WB regression model with the beta regression model. For this, we assumed that $\mathrm{confidence}∼\mathrm{WB}(\alpha_{i},\beta_{i})$ and $\mathrm{confidence}∼\mathrm{Beta}(\alpha_{i},\beta_{i})$ with

$$\begin{array}{cc} \log(\alpha_{i}) & =\gamma_{0}+\gamma_{1}\times {\mathrm{verdict}}_{i}+\gamma_{2}\times {\mathrm{conflict}}_{i}+\gamma_{3}\times {\mathrm{verdict}}_{i}\times {\mathrm{conflict}}_{i}\;\mathrm{and}\\ \log(\beta_{i}) & =\zeta_{0}+\zeta_{1}\times {\mathrm{verdict}}_{i}+\zeta_{2}\times {\mathrm{conflict}}_{i}+\zeta_{3}\times {\mathrm{verdict}}_{i}\times {\mathrm{conflict}}_{i}. \end{array}$$

Table 3 presents the AIC and BIC criteria for the two models. The WB model has the lower AIC and BIC, suggesting it should be preferred.

Table 4 shows the estimated parameters, standard errors, and significance levels for both models. Regarding the parameter vector γ associated with log(α), the WB model shows that both main effects, verdict (${\hat{\gamma }}_{1}=-0.3268$) and conflict (${\hat{\gamma }}_{2}=0.2854$), as well as their interaction term verdict:conflict (${\hat{\gamma }}_{3}=0.4450$), are statistically significant at the 5% level. In contrast, in the beta model, neither main effect in γ is statistically significant at the 5% level, leaving only the interaction term significant (${\hat{\gamma }}_{3}=0.2800$). Concerning the parameter vector ζ associated with log(β), verdict is statistically significant in both models (${\hat{\zeta }}_{1}=-0.3188$ for WB and ${\hat{\zeta }}_{1}=-0.3299$ for beta), while the interaction term verdict:conflict is significant only in the beta regression model (${\hat{\zeta }}_{3}=0.3163$). Overall, omitting the intercepts and using a 5% significance level, 4 of 6 coefficients are statistically significant in the WB model, whereas only 3 are significant in the beta model, showing that the proposed WB model captures predictor effects more effectively without relying heavily on higher-order interaction terms.

In addition, Table 5 displays the estimated mean, median, and mode for the response variable *confidence* across all experimental conditions. Both models yield consistent fitted central tendencies, showing that juror confidence drops when transitioning from a two-option to a three-option verdict setup or upon introducing conflicting evidence. However, the WB model provides distinct modal values under the presence of conflicting evidence, reflecting greater flexibility in capturing the distribution’s shape.

**Figure 2:**
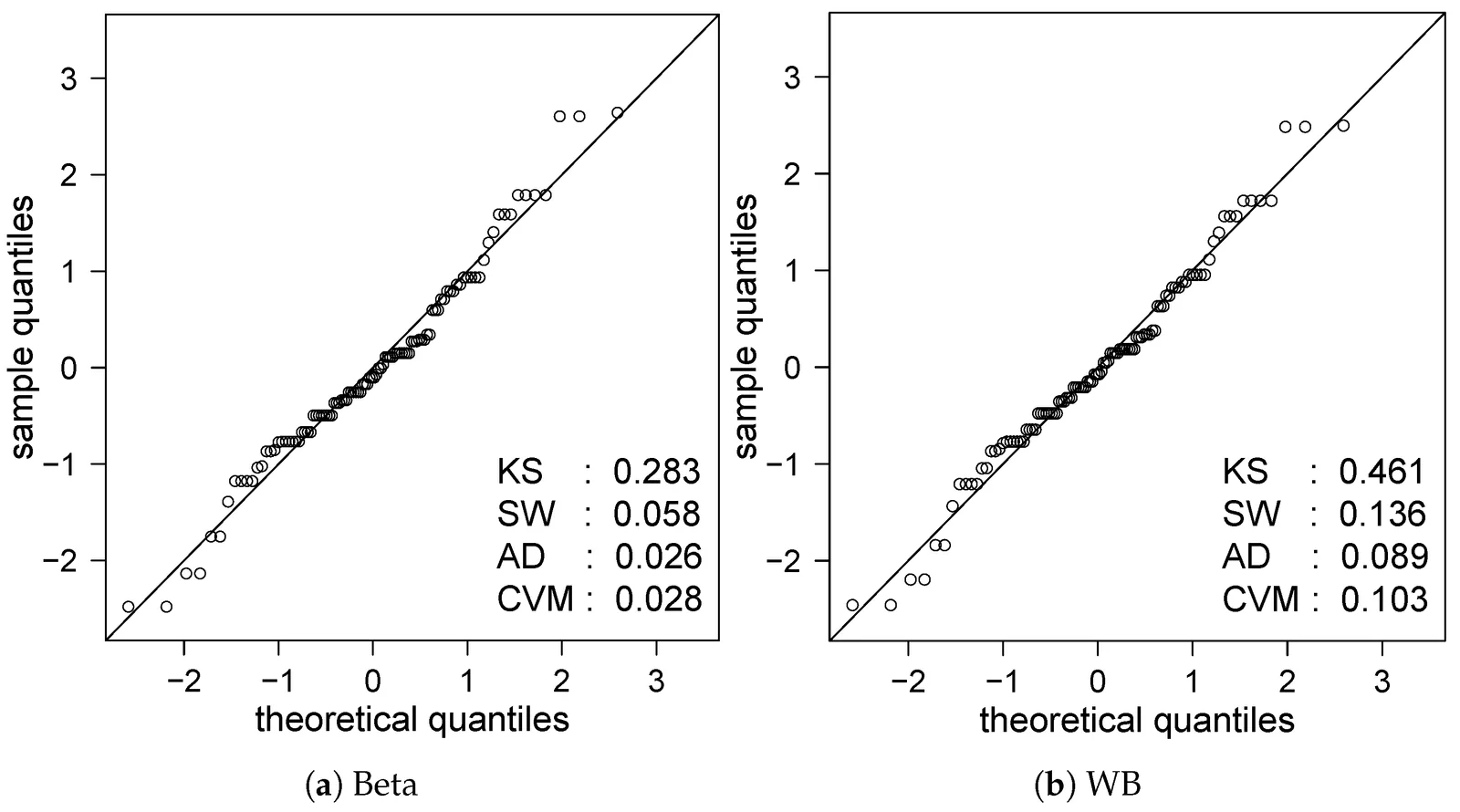
QR’s in the mock jurors dataset for beta and WB models.

On the other hand, Figure 2 presents the QR for the two models with the corresponding *p*-values for the KS, SW, AD, and CVM tests. Note that, with a 5% of significance, the 4 tests suggest that the standard normal distributions is suitable for the QR of the WB model; whereas 2 and none suggest the same for the beta model. Therefore, based on the QR, the WB model is more suitable than the beta model.

**Figure 3:**
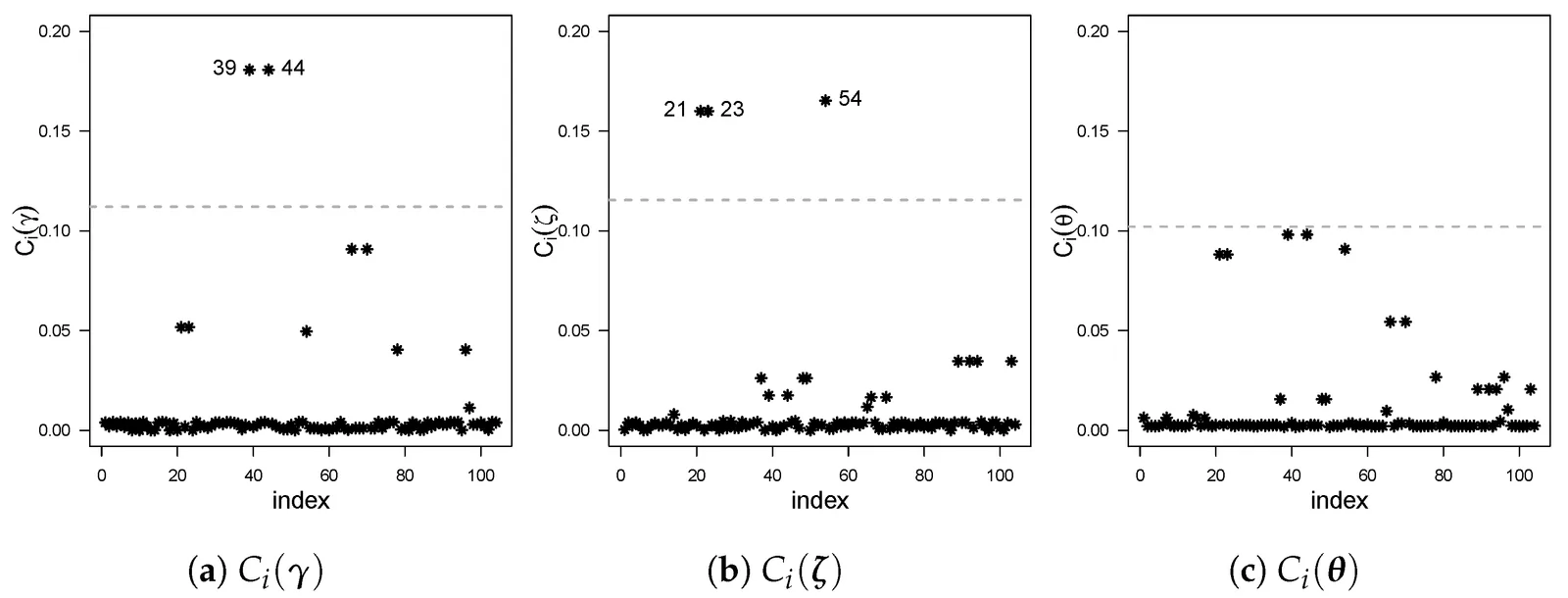

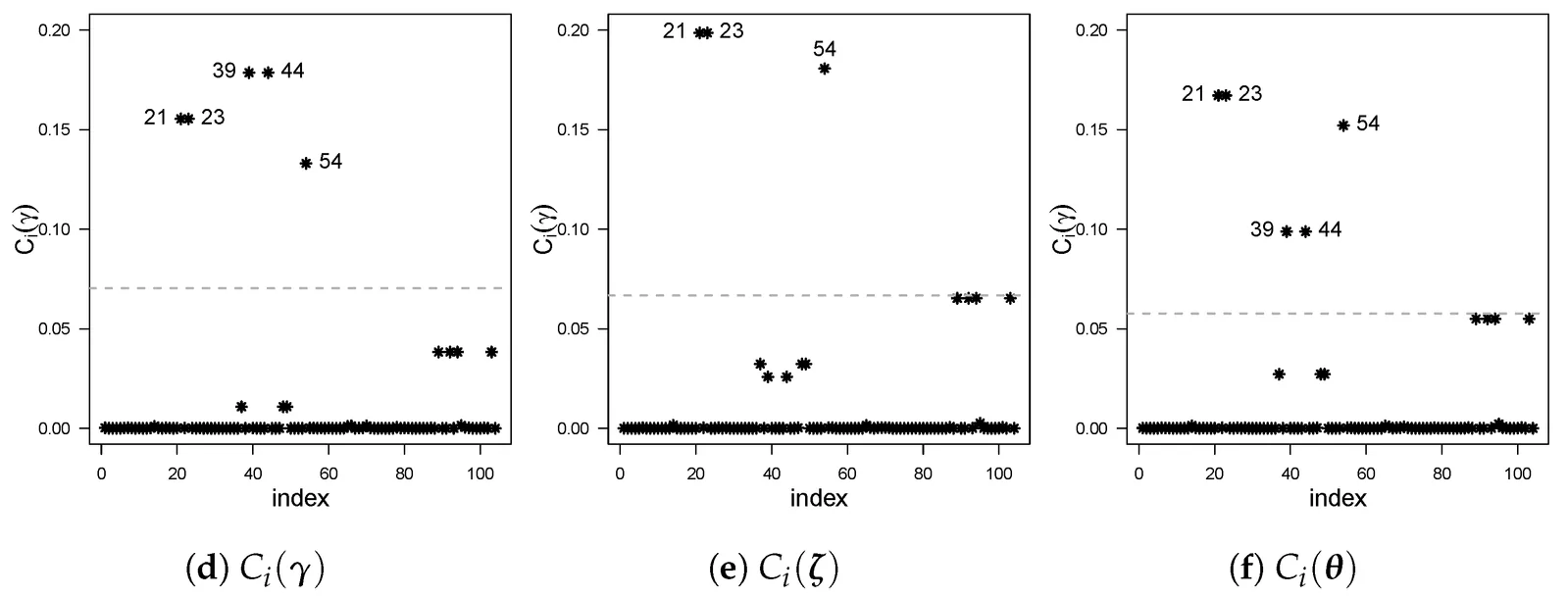
Index plot of $C_{i}$ in the mock jurors data set for WB model: (**a**,**b**,**c**) represent the perturbation of the case weights for γ, ζ and θ, respectively, and (**d**,**e**,**f**) represent the perturbation of the response for γ, ζ and θ, respectively.

**Figure 4:**
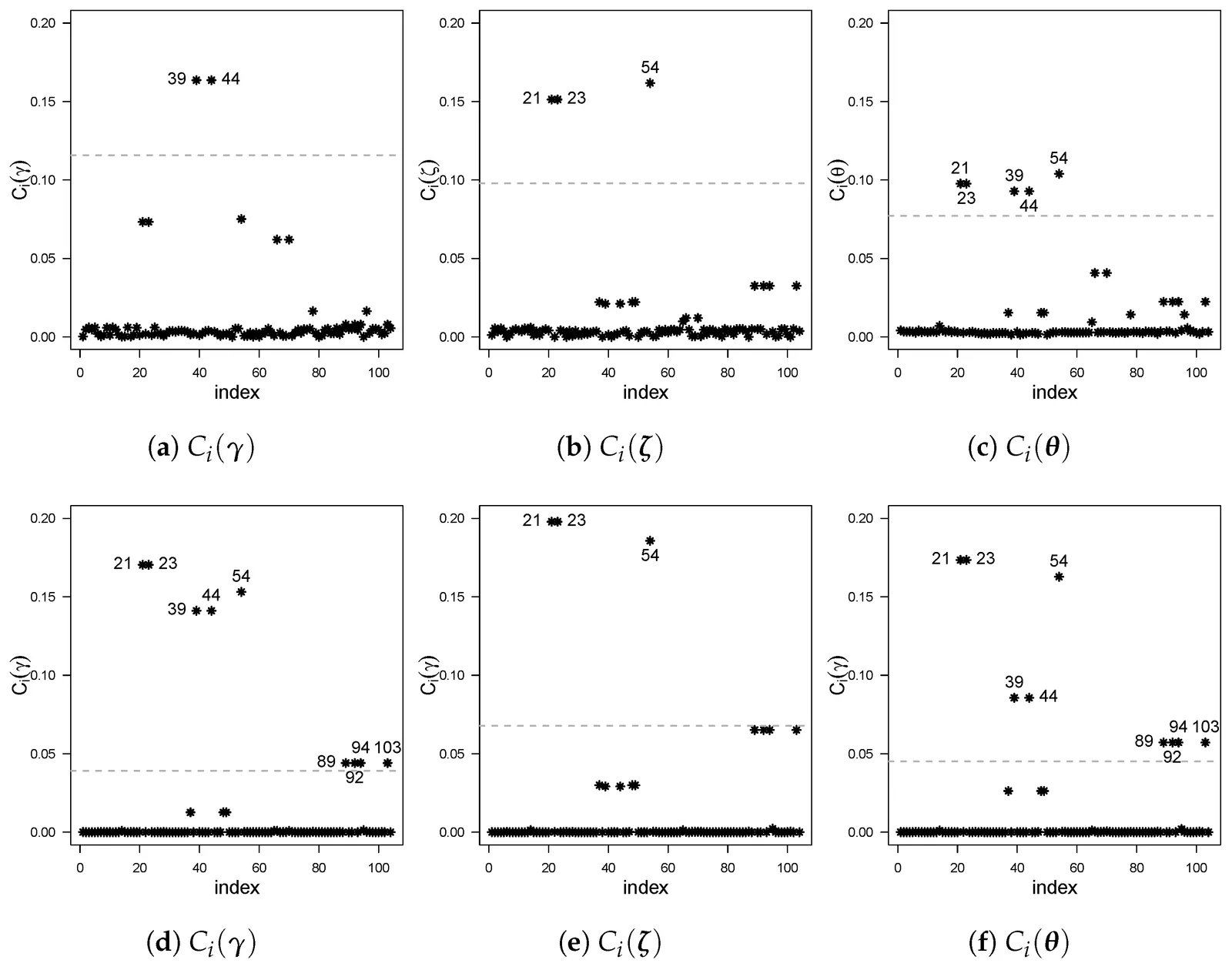
Index plot of $C_{i}$ in the mock jurors data set for beta model: (**a**,**b**,**c**) represent the perturbation of the case weights for γ, ζ and θ, respectively, and (**d**,**e**,**f**) represent the perturbation of the response for γ, ζ and θ, respectively.

Finally, Figure 3 and Figure 4 present the local influence results for the WB and beta models, respectively. Note that the observations #21, #23, #39, #44, and #54 are indicated as potentially influential for the WB model, whereas in addition to the referred observations, the individuals #89, #92, #94 and #103 are potentially influential for the Beta model, suggesting that the WB model is more robust than the Beta model for this data set.

## 8. Concluding Remarks

This paper has offered the WB distribution as a viable alternative to the beta distribution that shares many of the latter’s properties. The proposed distribution offers greater flexibility than the beta distribution in density shape. We provide a mathematical and statistical treatment of the new distribution, including a finite-mixture representation, stochastic representations, the CDF, and explicit expressions for its moments.. We also derive explicit expressions for the moments. Based on this new distribution, we have proposed a new regression model for modeling bounded data. We develop maximum likelihood estimation for the unknown parameters of the proposed regression model. The method demonstrated both good finite-sample and asymptotic results. The simulation results indicate satisfactory finite-sample performance of the maximum likelihood estimators, with biases and RMSEs decreasing as sample size increases, consistent with the expected asymptotic behavior. We obtained diagnostic tools to detect locally influential data in the ML estimates.

Finally, we fitted the proposed regression model to a real data set to show the potential of using the new methodology. Goodness-of-fit methods have demonstrated the suitable performance of the proposed model to these data in comparison to the beta distribution. However, the WB distribution is certainly not superior to the beta distribution in every way, i.e., the idea of this paper is not intended to promote the WB distribution as the best choice for all situations to analyze bounded data. The proposed regression model can be used for any application that involves unit interval data and is a natural strong competitor of the beta regression model. Users should always evaluate different distributions, with the proposed WB distribution as an additional alternative shown to be tractable and flexible across different data scenarios. A limitation of the current WB regression formulation is that it is not directly indexed by a central tendency parameter, such as the mean or median. Nevertheless, the adopted parameterization preserves the natural interpretation arising from the WB distribution’s stochastic representation.

In future work, we plan to extend the proposed regression model to accommodate data containing zeros and/or ones, explore alternative estimation methods, including Bayesian and EM-algorithm approaches, and develop a multivariate WB distribution as a generalization of the Dirichlet distribution for multivariate bounded data. A natural way of generating families of distributions [23] on some other support from a simple starting distribution with density *g* and CDF *G*, say, is to apply the quantile function $G^{-1}$ to a family of distributions on (0, 1). For the WB distribution, the resulting family has density

$$H\left(x\right)=\frac{\left(\alpha +\beta +1\right)}{\left(1+\alpha )(1+\beta \right)}\;\frac{g\left(x\right)G{\left(x\right)}^{\alpha -1}{\left(1-G(x)\right)}^{\beta -1}}{B(\alpha ,\beta )}\left\{(\alpha +\beta )\;G(x)\left[1-G(x)\right]+1\right].$$

## Figures and Tables

**Table 1:** Estimated bias (bias), root of the estimated RMSE, mean of the estimated SE and approximated 95% coverage probabilities.

		n=50				n=100				n=200				n=500			
Case	Parameter	Bias	SE	RMSE	CP	Bias	SE	RMSE	CP	Bias	SE	RMSE	CP	Bias	SE	RMSE	CP
I	$\gamma_{0}$	0.264	0.249	0.402	0.788	0.086	0.150	0.178	0.903	0.039	0.104	0.113	0.929	0.016	0.065	0.068	0.942
	$\gamma_{1}$	0.092	0.249	0.319	0.879	0.000	0.150	0.157	0.941	−0.003	0.104	0.106	0.947	0.001	0.065	0.065	0.950
	$\gamma_{2}$	−0.145	0.249	0.334	0.865	−0.014	0.150	0.157	0.937	−0.001	0.104	0.106	0.945	0.000	0.065	0.066	0.948
	$\gamma_{3}$	−0.113	0.249	0.319	0.882	−0.016	0.150	0.157	0.939	0.000	0.104	0.106	0.944	0.000	0.065	0.066	0.946
	$\zeta_{0}$	0.230	0.223	0.356	0.805	0.074	0.132	0.157	0.904	0.034	0.090	0.098	0.931	0.013	0.057	0.058	0.943
	$\zeta_{1}$	0.065	0.223	0.280	0.893	−0.005	0.132	0.140	0.935	−0.005	0.090	0.093	0.945	0.000	0.057	0.057	0.947
	$\zeta_{2}$	−0.123	0.223	0.296	0.880	−0.011	0.132	0.139	0.941	−0.002	0.090	0.092	0.946	0.001	0.057	0.056	0.951
	$\zeta_{3}$	−0.092	0.223	0.284	0.890	−0.012	0.132	0.139	0.938	0.000	0.090	0.092	0.946	0.001	0.057	0.057	0.948
II	$\gamma_{0}$	0.203	0.207	0.321	0.824	0.062	0.121	0.140	0.910	0.026	0.083	0.088	0.935	0.010	0.052	0.054	0.945
	$\gamma_{1}$	0.082	0.207	0.262	0.897	0.007	0.121	0.126	0.940	0.001	0.083	0.085	0.941	0.000	0.052	0.052	0.950
	$\gamma_{2}$	−0.122	0.207	0.279	0.877	−0.015	0.121	0.128	0.937	−0.002	0.083	0.084	0.946	0.000	0.052	0.052	0.952
	$\gamma_{3}$	−0.077	0.207	0.261	0.898	−0.006	0.121	0.126	0.941	0.002	0.083	0.084	0.947	0.002	0.052	0.053	0.947
	$\zeta_{0}$	0.368	0.302	0.533	0.737	0.134	0.201	0.261	0.869	0.060	0.141	0.162	0.909	0.025	0.088	0.093	0.931
	$\zeta_{1}$	0.037	0.302	0.387	0.870	−0.039	0.201	0.226	0.913	−0.025	0.141	0.150	0.933	−0.009	0.088	0.090	0.940
	$\zeta_{2}$	−0.105	0.302	0.401	0.860	0.018	0.201	0.224	0.918	0.019	0.141	0.149	0.932	0.007	0.088	0.090	0.941
	$\zeta_{3}$	−0.203	0.302	0.438	0.833	−0.063	0.201	0.235	0.905	−0.024	0.141	0.150	0.929	−0.009	0.088	0.090	0.940

**Table 2:** Percentage of times in which the respective test does not reject the hypothesis of normality for the QR. The log-likelihood row indicates the percentage of times the corresponding model provides a higher log-likelihood.

True			n=50		n=100		n=200		n=500	
Model	Case	Criteria	WB	BE	WB	BE	WB	BE	WB	BE
WB	I	KS	1.00	1.00	1.00	1.00	1.00	1.00	1.00	0.99
		SW	0.95	0.94	0.95	0.93	0.95	0.89	0.95	0.79
		AD	0.94	0.93	0.95	0.92	0.95	0.89	0.95	0.80
		CVM	0.94	0.93	0.95	0.92	0.95	0.89	0.95	0.81
		Log-likelihood	0.46	0.54	0.56	0.44	0.63	0.37	0.74	0.26
	II	KS	1.00	1.00	1.00	1.00	1.00	1.00	1.00	0.99
		SW	0.95	0.94	0.95	0.94	0.96	0.92	0.96	0.86
		AD	0.94	0.94	0.95	0.94	0.95	0.92	0.95	0.87
		CVM	0.94	0.94	0.95	0.93	0.95	0.92	0.95	0.88
		Log-likelihood	0.63	0.37	0.66	0.34	0.70	0.30	0.80	0.20
BE	I	KS	1.00	1.00	1.00	1.00	1.00	1.00	0.99	1.00
		SW	0.93	0.95	0.93	0.96	0.88	0.95	0.75	0.95
		AD	0.92	0.94	0.91	0.95	0.88	0.95	0.76	0.95
		CVM	0.92	0.94	0.92	0.95	0.88	0.95	0.77	0.95
		Log-likelihood	0.33	0.67	0.32	0.68	0.28	0.72	0.19	0.81
	II	KS	1.00	1.00	1.00	1.00	1.00	1.00	0.99	1.00
		SW	0.94	0.95	0.94	0.95	0.92	0.96	0.87	0.96
		AD	0.93	0.94	0.92	0.95	0.91	0.95	0.87	0.95
		CVM	0.93	0.94	0.92	0.95	0.92	0.95	0.88	0.95
		Log-likelihood	0.47	0.53	0.42	0.58	0.33	0.67	0.22	0.78

**Table 3:** Criteria for mock jurors data set for Beta and WB models.

	Beta	WB
Log-likelihood	40.12	41.69
AIC	−64.23	−67.38
BIC	−43.08	−46.23

**Table 4:** Estimated parameters for beta and WB models in the mock jurors dataset.

		Beta			WB		
	Parameter	Estimate	SE		Estimate	SE	
γ	(Intercept)	0.9124	0.1040	***	0.8175	0.1426	***
	verdict	0.0050	0.1040		−0.3268	0.1426	*
	conflict	0.1686	0.1040		0.2854	0.1426	*
	verdict:conflict	0.2800	0.1040	**	0.4450	0.1426	**
ζ	(Intercept)	1.1733	0.1278	***	−0.2319	0.1228	.
	verdict	−0.3299	0.1278	**	−0.3188	0.1228	*
	conflict	0.2196	0.1278	.	0.0823	0.1228	
	verdict:conflict	0.3163	0.1278	*	0.1072	0.1228	

. Significant at the 10% significance level. * Significant at the 5% significance level. ** Significant at the 1% significance level. *** Significant at the 0.1% significance level.

**Table 5:** Estimated mean, median, and mode for confidence in the mock jurors data set based on the Beta and WB models.

		Beta			WB		
Verdict	Conflict	Mean	Median	Mode	Mean	Median	Mode
two-option	no	0.7969	0.8477	0.9999	0.7952	0.8384	0.9999
three-option	no	0.5707	0.6002	0.9999	0.5719	0.5930	0.9999
two-option	yes	0.6104	0.6358	0.7961	0.6136	0.6321	0.7071
three-option	yes	0.5696	0.5896	0.7967	0.5722	0.5868	0.6569

## Data Availability

The data set used is included in the betareg package. https://cran.r-project.org/web/packages/betareg/index.html, accessed on 9 October 2026.

## Appendix A

**Proof of Theorem 1.** Since for 0<z<1, the sign of the derivative of the density given in (4) is completely determined by the sign of $p_{3}$. We first note that

$$p_{3}\left(0;\alpha ,\beta \right)=\alpha -1\;\mathrm{and}\;p_{3}\left(1;\alpha ,\beta \right)=1-\beta .$$

1. Suppose first that α<1 and β<1. In this case, $p_{3}\left(0;\alpha ,\beta \right)<0\;\mathrm{and}\;p_{3}\left(1;\alpha ,\beta \right)>0.$ Hence, by the intermediate value theorem, $p_{3}$ has at least one zero in (0,1). Since $p_{3}$ is a cubic polynomial, the number of zeros in (0,1), counting multiplicities, is either one or three. If there is only one zero, the sign of $p_{3}$ changes from negative to positive, and therefore $f_{Z}$ is decreasing-increasing, which, together with $\lim_{z\to 0^{+}}f_{Z}\left(z;\alpha ,\beta \right)=\lim_{z\to 1^{-}}f_{Z}\left(z;\alpha ,\beta \right)=\infty ,$ gives a U-shaped density. If there are three distinct zeros $0<z_{1}<z_{2}<z_{3}<1$, the sign pattern of the cubic polynomial is −,+,−,+, and consequently the density is decreasing-increasing-decreasing-increasing. This proves part 1. 2. Suppose that α<1<β. Let $c_{1}=\left(\alpha +\beta \right)\left(\alpha -1\right)+2.$ We have $p_{3}\left(0\right)=\alpha -1<0,\;p_{3}\left(1\right)=1-\beta <0,$ and $\lim_{z\to \infty }p_{3}\left(z\right)=+\infty .$ If $c_{1}⩽0$, the signs of the coefficients of $p_{3}$ are +,−,−,−. Hence, by Descartes’ rule of signs, $p_{3}$ has exactly one positive root. Since $p_{3}\left(1\right)<0$ and $p_{3}\left(z\right)\to +\infty$ as z→∞, this root belongs to (1,∞). Therefore, $p_{3}\left(z\right)<0,\;z\in \left(0,1\right),$ and $f_{Z}$ is decreasing on (0,1). Now suppose that $c_{1}>0$. In this case, the signs of the coefficients of $p_{3}$ are +,−,+,−. Moreover, $p_{3}\left(-z\right)=-{\left(\alpha +\beta \right)}^{2}z^{3}-\left(\alpha +\beta \right)\left(2\alpha +\beta \right)z^{2}-c_{1}z+\alpha -1<0,\;z>0.$ Thus, $p_{3}$ has no negative roots. If Δ(α,β)>0, then $p_{3}$ has three distinct real roots. Since $p_{3}$ has no negative roots, all three roots are positive. Because one root belongs to (1,∞), while $p_{3}\left(0\right)<0,\;p_{3}\left(1\right)<0,$ the other two roots belong to (0,1). Hence, $0<z_{1}<z_{2}<1<z_{3}.$ Consequently, the sign of $p_{3}$ on (0,1) is −,+,−, and therefore $f_{Z}$ has a decreasing–increasing–decreasing shape. If Δ(α,β)<0, then $p_{3}$ has a unique real root. Since $p_{3}\left(1\right)<0$ and $p_{3}\left(z\right)\to +\infty$, this root belongs to (1,∞). Hence, $p_{3}\left(z\right)<0,\;z\in \left(0,1\right),$ and $f_{Z}$ is decreasing on (0,1). Finally, if Δ(α,β)=0, then $p_{3}$ has a multiple real root. Since $p_{3}$ has no negative roots and $p_{3}\left(0\right)<0$, $p_{3}\left(1\right)<0$, any root in (0,1) must have even multiplicity and therefore does not change the sign of $p_{3}$. Thus, $p_{3}\left(z\right)⩽0,\;z\in \left(0,1\right),$ and $f_{Z}$ is non-increasing on (0,1). This proves part 2. 3. Now, suppose that α>1>β. Since 1−Z∼WB(β,α) whenever Z∼WB(α,β), this case follows from part 2 by the transformation z↦1−z. Indeed, the corresponding cubic polynomials satisfy $p_{3}\left(z;\beta ,\alpha \right)=-p_{3}\left(1-z;\alpha ,\beta \right).$ Hence, their roots are related by the transformation z↦1−z. Moreover, the discriminant is invariant under this transformation, so that Δ(α,β)=Δ(β,α). Therefore, if Δ(α,β)>0, the density of *Z* has an increasing-decreasing-increasing shape, whereas if Δ(α,β)⩽0, the density is non-decreasing. This proves part 3. 4. By Descartes’ rule of signs, the equation $p_{3}\left(z;\alpha ,\beta \right)=0$ has either two or zero positive roots, since the coefficients of $p_{3}$ have signs +,−,+,+. Moreover, $p_{3}\left(0;\alpha ,\beta \right)=\alpha -1>0,\;p_{3}\left(1;\alpha ,\beta \right)=1-\beta <0,$ and $\lim_{z\to \infty }p_{3}\left(z;\alpha ,\beta \right)=+\infty .$ Therefore, by the Intermediate Value Theorem, there is at least one root in (0,1) and at least one root in (1,∞). Hence, $p_{3}$ has at least two positive roots. Since, by Descartes’ rule of signs, it has at most two positive roots, there are exactly two positive roots: one in (0,1) and one in (1,∞). In particular, the root in (0,1) is unique. It follows from (4) that $f_{Z}\left(z;\alpha ,\beta \right)$ has exactly one critical point in (0,1). Since $\lim_{z\to 0^{+}}f_{Z}\left(z;\alpha ,\beta \right)=\lim_{z\to 1^{-}}f_{Z}\left(z;\alpha ,\beta \right)=0,$ and $f_{Z}^{\prime }\left(z;\alpha ,\beta \right)>0\;\mathrm{before}\;\mathrm{the}\;\mathrm{critical}\;\mathrm{point}$ and $f_{Z}^{\prime }\left(z;\alpha ,\beta \right)<0\;\mathrm{after}\;\mathrm{the}\;\mathrm{critical}\;\mathrm{point},$ this critical point is the unique mode of the WB density. Therefore, the WB PDF is unimodal. This proves part 4. □
